# Cirrhosis related functionality characteristic of the fecal microbiota as revealed by a metaproteomic approach

**DOI:** 10.1186/s12876-016-0534-0

**Published:** 2016-10-04

**Authors:** Xiao Wei, Shan Jiang, Yuye Chen, Xiangna Zhao, Huan Li, Weishi Lin, Boxing Li, Xuesong Wang, Jing Yuan, Yansong Sun

**Affiliations:** 1 Institute of Disease Control and Prevention, Academy of Military Medical Sciences, No. 20 Dongda Street, Fengtai District, 100071 Beijing, China; 2 Hospital of Traditional Chinese Medicine, Liquan, 713200 Shanxi China

**Keywords:** Cirrhosis, Metaproteome, Fecal microbiota, BCAA

## Abstract

**Background:**

Intestinal microbiota operated as a whole and was closely related with human health. Previous studies had suggested close relationship between liver cirrhosis (LC) and gut microbiota.

**Methods:**

To determine the functional characteristic of the intestinal microbiota specific for liver cirrhosis, the fecal metaproteome of three LC patients with Child-Turcotte-Pugh (CTP) score of A, B, and C, and their spouse were first compared using high-throughput approach based on denaturing polyacrylamide gel electrophoresis and liquid chromatography–tandem mass spectrometry in our study.

**Results:**

A total of 5,020 proteins (88 % from bacteria, 12 % form human) were identified and annotated based on the GO and KEGG classification. Our results indicated that the LC patients possessed a core metaproteome including 119 proteins, among which 14 proteins were enhanced expressed and 7 proteins were unique for LC patients compared with the normal, which were dominant at the function of carbohydrate metabolism. In addition, LC patients have unique biosynthesis of branched chain amino acid (BCAA), pantothenate, and CoA, enhanced as CTP scores increased. Those three substances were all important in a wide array of key and essential biological roles of life.

**Conclusions:**

We observed a highly comparable cirrhosis-specific metaproteome clustering of fecal microbiota and provided the first supportive evidence for the presence of a LC-related substantial functional core mainly involved in carbohydrate, BCAA, pantothenate, and CoA metabolism, suggesting the compensation of intestinal microbiota for the fragile and innutritious body of cirrhotic patients.

**Electronic supplementary material:**

The online version of this article (doi:10.1186/s12876-016-0534-0) contains supplementary material, which is available to authorized users.

## Background

Human intestinal tract is colonized by a complex and intimate microbial community that is maintained from early to late adulthood [1]. Human intestinal microbiota was an important organ which has been neglected for a long time. A lot of evidence has suggested that intestinal microbiota works as a whole and plays an important role in human health [2].

Cirrhosis is a condition in which the liver failed to work properly due to long-term damage. The anatomy and physiological functions of liver and intestinal microbiota share a close relationship as a result of enterohepatic circulation. As the cirrhosis worsen, it was usually accompanied with intestinal inflammation, which may affect the prognosis significantly [3]. Previous studies had revealed the important influence of gut microbiota dysbiosis on the complications of advanced liver cirrhosis (such as spontaneous bacterial peritonitis and hepatic encephalopathy) [4] and on the induction and progress of liver damage at early cirrhosis (such as alcoholic hepatitis and nonalcoholic fatty liver disease) [5]. It has been identified that extensive differences in the microbiota community and metabolic potential existed in the fecal microbiota of cirrhotic patients, and the prevalence of potentially pathogenic bacteria, such as *Enterobacteriaceae* and *Streptococcaceae*, with the reduction of beneficial populations such as *Lachnospiraceae* in patients with cirrhosis may affect prognosis [3, 6]. In addition, The functional diversity was significantly reduced in the fecal microbiota of cirrhotic patients compared with in the controls. At the module or pathway levels, the fecal microbiota of the HBLC patients showed enrichment in the metabolism of glutathione, branched-chain amino acid, nitrogen, and lipid, whereas there was a decrease in the level of aromatic amino acid, bile acid and cell cycle related metabolism [3]. These high-throughput analyses lay the groundwork for predicting the protein potential of the intestinal microbiota. However, the functional and metabolic associations between intestinal microbiota and cirrhosis in humans are still lacking.

The rapidly increasing catalogue of proteins from intestinal origin provides a platform for high-throughput functional characterization [7]. In the past 20 years, more and more interests were focused on fecal microbiota, partly due to the fast emerging sequencing technique. Given that proteins are much more actual and stable, proteome-based analyses can be expected to provide a more accurate and real view of the functionality of the intestinal microbiota. The LC-MS/MS method has a higher level of proteome coverage, and is more efficient and accurate to analyze differential global protein expression quantitatively and qualitatively. So far there have been several reports related with the use of metaproteomics to characterize complex bacterial ecosystems [8, 9]. 2015, Hernández E et al. collected intestinal microbiota proteins from two adult patients receiving 14-days β-lactam therapy, seven obese and five lean adolescents, followed by metaproteome measurements, to assess the functional differences and metabolic effects in human gastrointestinal tract related with antibiotic treatment and obesity [10]. 2012, Pérez-Cobas AE carried out the multi-omic research on fecal samples from one patient subjected to a β-lactam intravenous therapy, which suggested that antibiotics may ultimately alter the energy metabolism balance in human gastrointestinal tract [11]. 2012, faecal samples were collected from 3 healthy female subjects over a period of six to twelve months to research the composition and stability of human intestinal metaproteome [12]. 2006, Klaassens ES et al. collected fecal samples from two infants at different ages to characterize the complex intestinal bacterial structure, and illustrated the feasibility of metaproteomic approach in analyzing complex mciroecosystem [13].

In this work, human intestinal microbiota was regarded as a whole to analyze. A high-throughput LC-MS/MS measurement were used to detect the metaproteomic changes in the intestinal microbiota of LC patients. This is the first culture-independent metaproteomic analysis in the gastrointestinal tract from cirrhotic patients. Our findings may provide a more comprehensive understanding of fecal microbiota in patients with cirrhosis, and generate novel perspectives on the progress and prognosis of cirrhosis.

## Methods

### Fecal samples

The Child-Turcotte-Pugh (CTP) scoring system was used to assess the severity of cirrhosis. Three LC patients (CTP score of A, B, C) and their corresponding spouse, in the age range of 50–60 and with a body mass index (BMI) = 18.5–24.9 kg m^−2^, were enrolled in this study. Cirrhosis was diagnosed according to the gold standard of biopsy in all patients. Cases that had other complications (such as peritonitis or hepatic encephalopathy) were excluded in this study. All healthy individuals had normal liver biochemistry test results with no evidence of hepatic or other diseases. None of the subjects had food preferences, or received antibiotics, probiotics, steroids or other hormones (including oral, intramuscular or intravenous injection) for at least 3 months before enrolment. Each object was asked to provide a fresh stool sample, which were subjected to metaproteome extraction immediately. This study was approved by the Institutional Review Board of Affiliated Hospital of Academy of Military Medical Sciences. All participants signed an informed consent form prior to entering the study. The study conformed to the ethical guidelines of the 1975 Declaration of Helsinki.

### Protein extraction

Faecal samples were resuspended in PBS and vortexed to homogenize the sample. Proteins were precipitated in pre-chilled TCA (trichloroacetic acid)-acetone (50 g TCA dissolved in 500 ml acetone) at -20 °C for 2 h. After centrifugation at 20,000 × g for 30 min, the protein pellet was washed with pre-chilled acetone and ultrasonicated in lysis buffer (8 M urea, 30 mM HEPES (4-(2-Hydroxyethyl)-1-piperazineethanesulfonic acid), 1 mM PMSF (Phenylmethanesulfonyl fluoride), 2 mM EDTA (Ethylene Diamine Tetraacetie Acid), 10 mM DTT (DL-Dithiothreitol)). After centrifugation at 20,000 × g for 30 min, the supernatant were reduced with 10 mM DTT at 56 °C for 1 h, and alkylated with 55 mM IAM (iodoacetamide) at room temperature for 1 h in the dark. The treated proteins were precipitated in pre-chilled acetone at −20 °C for 3 h. After centrifugation at 20,000 × g for 30 min, the protein pellet was resuspended and ultrasonicated in pre-cooled 50 % TEAB (tetraethylammonium bromide) buffer with 0.1 % SDS, and then centrifuged at 20,000 × g to pellet the undissolved substance. The protein concentrations were measured by Bradford assay.

### 1 D gel electrophoresis and in-gel protein digestion

To reduce the complexity of the protein extract, a 1D gel fractionation according to molecular weight was carried out. Equal volumes of protein solutions were mixed with 4 × Laemmli buffer and Biorad reducing agent and run on NuPAGE 4–12 % Bis-Tris gels (Invitrogen) at a constant voltage of 80 V for 20 min followed by 200 V for 30 min. Gels were stained with PageBlue (Fermentas). Gel pieces were washed and proteins reduced, alkylated and tryptically digested overnight as described previously [14].

### LC-MS/MS measurements

Peptide samples were analyzed by LC-MS/MS on a quadrupole-Orbitrap mass spectrometer (Q-Exactive; Thermo Fisher, Germany) equipped with a 15 cm (length) by 75 μm (inside diameter) column packed with 5 μm C18 medium (150A, Thermo Fisher) which was remained at 21 °C throughout the analysis. Mobile phase A was MilliQ water with 0.1 % (*v/v*) formic acid. Mobile phase B was 99.9 % (*v/v*) acetonitrile and 0.1 % acetic acid. Gradient was run from 0 % B to 30 % B over 40 min and then to 80 % B for 15 min. An electrospray voltage of 1.8 kV was applied. By data-dependent acquisition, the mass spectrometer was programmed to acquire tandem mass spectra from the top 20 ions in the full scan from 350 to 2,000 m/z. Dynamic exclusion was set to 15 s, singly charged ions were excluded, the isolation width was set to 2 m/z. The full MS resolution was set to 70,000, and the MS/MS resolution to 17,500. Normalized collision energy was set to 28, automatic gain control to 1e6, maximum fill MS to 20 ms, maximum fill MS/MS to 60 ms, and the under fill ratio to 0.1 %. Each sample was repeated triply.

### Protein identificaton

Peptide identification were performed using Mascot v2.3.01 (Matrix Science Ltd.) (http://www.matrixscience.com) licensed in-house (http://www.proteomics.cn) [15]. Monoisotopic peptide masses were used to search the databases, allowing a peptide mass accuracy of 30 ppm and fragment ion tolerance of 0.2 Dalton. Both methionine oxidation and cysteine carboxyamidomethylation were considered in the process. For protein identification, peptide masses were searched against the publically available database for Uniprot-Human database and Uniprot-Bacteria database. For unambiguous identification of proteins, more than five peptides must be matched and the sequence coverage must be greater than 15 %.

### Bioinformatic analysis

Microbial proteins which had at least one peptide identification in each individual were defined. Functional classification of identified proteins was performed by BLASTPGP [16] searching against the databases of Cluster of Orthologous Groups database (COGs, ftp://ftp.ncbi.nih.gov/pub/COG/COG2014/static/lists/listCOGs.html) [17]. The COG associated with the best BLAST hit (≤1E-10 cutoff) was assigned to the query protein. The identified COGs were mapped on the KEGG metabolic pathways and visualized with the online application of iPath [18]. The spectra those proteins were identified with were taken to sum on COG level per all measurements and were used to rank the COGs according to spectra number. Sequences which did not hit any specific COG were subjected to BLAST search against the NCBI non-redundant database. The cellular localizations of the proteins were predicted by PSORTb version 3.0 [19]. Prediction of signal peptides was carried out with SignalP Version 4.1 [20]. The metaproteomic data from three LC patients (CTP score of A, B, C) were compared with their corresponding spouse, respectively.

## Results

### Identification of proteins and metabolic pathways in the fecal metaproteome

**Fig. 1:**
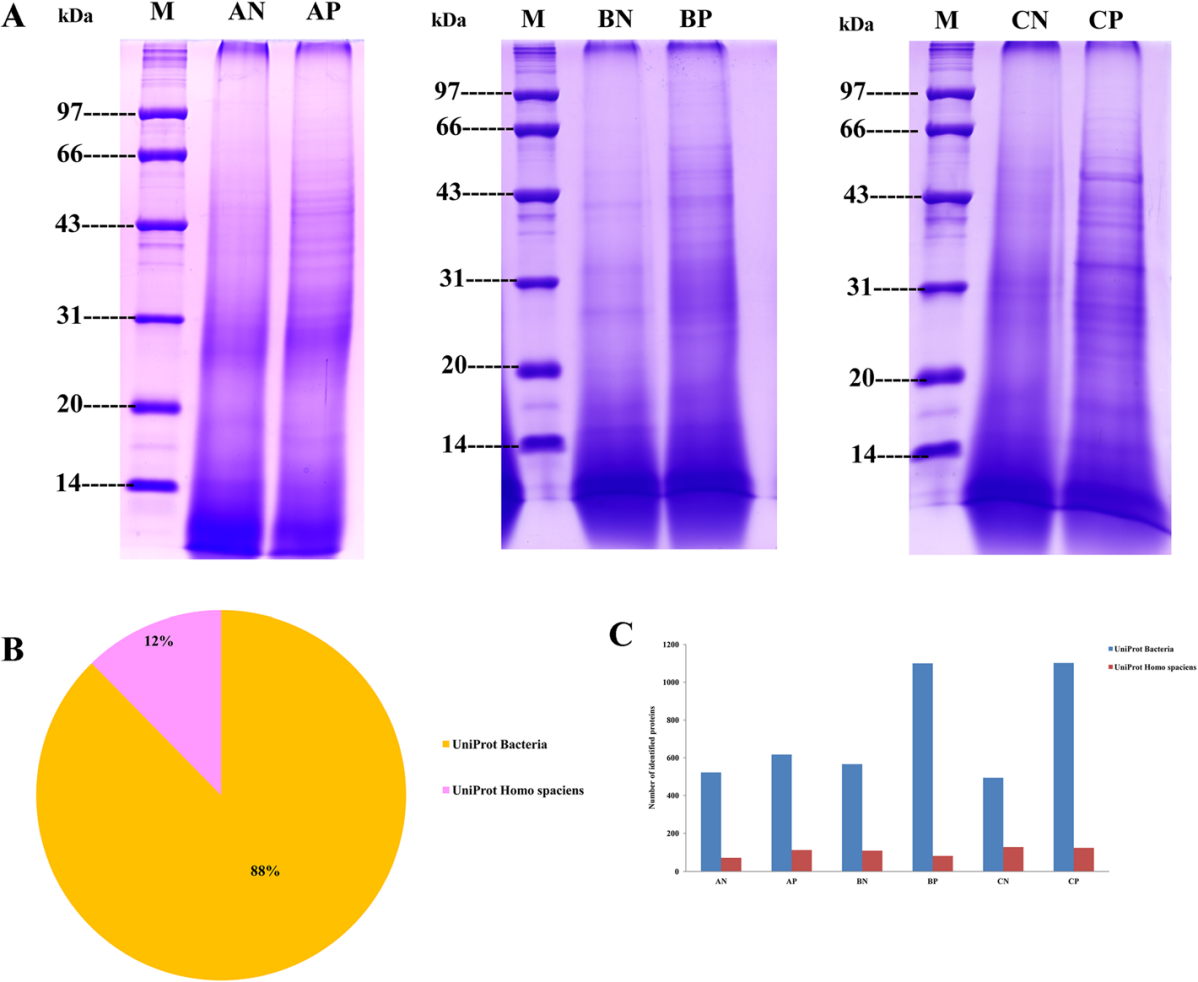
Electrophoresis maps and annotation results of the fecal metaproteome from six subjects. **a** The 1D-gel showing the protein pattern from fecal metaproteome of six subjects. **b** Proportion of proteins allocated to uniprot-bacteria and uniprot-human databases. In this study, a total of 5,020 proteins were identified with two or more peptide identifications, in which 88 % from bacteria proteins and 12 % from human proteins. **c** Number of proteins allocated to uniprot-bacteria and uniprot-human databases in each sample

Three LC patients with CTP score of A, B, C (coded as AP, BP, CP) and their corresponding spouse (coded as AN, BN, CN) were enrolled in this study. Characteristics of the subjects were given in Table 1. Unprocessed fecal material from those subjects were used not only to reappear the intestinal microbial proteome characteristics, but also to allow detection of human proteins. Fecal metaproteome were extracted and subjected to 1D-gel (Fig. 1a). A total of over 100,000 spectra were generated by the collection of LC-MS/MS. The raw data has been uploaded to the public websites (http://www.iprox.org/my/index, user ID: reviewer 719, password: 6s6kd6lp). In this study, a total of 5,020 proteins were identified with two or more peptide identifications, including 4,401 (88 %) from bacteria proteins and 619 (12 %) from human proteins (Fig. 1b). Results from the annotation to Uniprot-Bacteria database suggested that almost equal and 600 proteins were identified from AP and AN, and 1100 and 1102 proteins identified from BP and CP respectively, almost twice more than their controls. Results of the fecal metaproteome assigned to Uniprot-Human database suggested a relatively stable expression and an average of 100 proteins were identified from each sample (Fig. 1c). Annotation results of bacterial proteins and human proteins from each sample were shown in Additional file 1: Table S1 and Additional file 2: Table S2. To retrieve further functional information, we annotated these proteins based on the GO and KEGG classification and the overall number of proteins identified were shown in Table 2.

**Table 1:** Characteristics of the patients and controls

Terms	AP	AN	BP	BN	CP	CN
Age	52	50	59	56	51	50
Gender	Male	Female	Male	Female	Male	Female
BMI index	24.1	24.3	25.3	25.1	24.9	25.0
Total bilirubin (μmol/L)	6.6	—	11.8	—	36.3	—
Albumin (g/L)	37.1	—	30.5	—	26.2	—
Stage of hepatic encephalopathy	0	—	0	—	1	—
Ascites degree	No	—	Moderate	—	Severe	—
Prothrombin time prolonged (seconds)	0	—	2	—	2.1	—
CTP	A	—	B	—	C	—

**Table 2:** Overall number of proteins identified from patients and the normal

	Uniprot-Bacteria		Uniprot-Human	
	Patients	Normal	Patients	Normal
Total Protein Number	2,819	1,582	314	305
GO (Cellular Component)				
Number of GO^a^	93	81	171	190
Number of Proteins^b^	1,213	594	87	98
GO (Biological Process)				
Number of GO^a^	1,004	781	867	1,082
Number of Proteins^b^	1,890	954	75	92
GO (Molecular Function)				
Number of GO^a^	861	596	230	248
Number of Proteins^b^	2,173	1,092	91	110
KEGG				
Number of KEGG^a^	127	100	106	123
Number of Proteins^b^	1,697	865	80	80

^a^Number of GO terms or KEGG pathways identified

^b^Number of proteins identified and assigned to corresponding GO terms or KEGG pathways

### Core metaproteome specific for LC patients

**Fig. 2:**
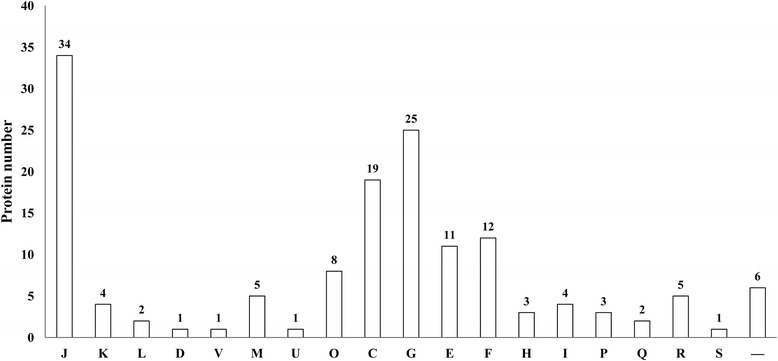
Functional category of the common and core metaproteome from LC patients. The common and core metaproteome could be grouped into 19 COGs and the most predominant functional categories were J, G, C, F, and E. Categories were taken from the TIGR-CMR (www.tigr.org) and the abbreviation was used to mark the categories. J, translation; K, transcription; L, replication, recombination, and repair; D, cell cycle control, mitosis, and meiosis; V, defense mechanisms; M, cell wall/membrane biogenesis; U, intracellular trafficking and secretion; O, post-translational modification, protein turnover, chaperones; C, energy production and conversion; G, carbohydrate transport and metabolism; E, amino acid transport and metabolism; F, nucleotide transport and metabolism; H, coenzyme transport and metabolism; I, lipid transport and metabolism; P, inorganic ion transport and metabolism; Q, secondary metabolite biosynthesis, transport, and catabolism; R, general function prediction only; S, function unknown; —, not in Clusters of Orthologous Groups (COG)

To obtain the LC-related common microbial core proteome, we selected proteins that were found in all the three patients. A total of 119 proteins fulfilled this criterion and their description and functional characteristics were shown in Additional file 3: Table S3. The common and core metaproteome could be grouped into 18 COGs and the most predominant functional categories were J (translation), G (carbohydrate transport and metabolism), C (energy production and conversion), F (nucleotide transport and metabolism), and E (amino acid transport and metabolism) (Fig. 2). Nearly 19 % proteins could be assigned to carbohydrate transport and metabolism, and 8 % assigned to amino acid transport and metabolism, reflecting the high metabolic activity of the intestinal microbiota from LC patients. Among those 119 core proteins, 14 proteins enhanced their expression levels and 7 proteins were specific for LC patients compared with the normal, which were described as below.

### Differential fecal microbial metabolism and proteins in LC patients

Fourteen KEGG pathway maps were detected to have different metabolic capacities in the fecal microbiota between patients and the normal, including eleven pathways enhanced and three pathways weakened in patients (Additional file 4: Table S4). In the same metabolic pathway, different proteins were identified in different samples, suggesting that the intestinal microbiota have abundant species diversity and protein complexity. In five metabolic pathways, we detected same proteins in different samples, most of which were assigned to carbohydrate metabolism. One of the most predominant pathways was glycolysis/gluconeogenesis, showing high redundancies among microbiota; all the glycolytic/gluconeogenic enzymes could be identified in patients, highlighting the enhanced material metabolism capacity in intestinal microbiota of LC patients.

Fourteen proteins were detected to have enhanced expression level in all the three patients compared with the normal (Table 3), which could be grouped into four COGs (J: Translation, G: Carbohydrate transport and metabolism, E: Amino acid transport and metabolism, O: Posttranslational modification, protein turnover, chaperones), suggesting the enhanced material transport and metabolism function in fecal microbiota from LC patients.

**Table 3:** Characteristics of proteins with enhanced expression level in the fecal microbiota from LC patients or specific for patients’ intestinal microbiota compared with the normal

No.	Description	Gene name	Length	MW [Da]	KO	COG	Subcellular location	Signal peptide prediction	GO	Functonal Category^a^
Proteins with enhanced expression level in the fecal microbiota from LC patients										
1	Chaperone protein DnaK	dnaK	620	66,266	k04043	COG0443	Cytoplasmic	Absence	GO:0006457 GO:0000166 GO:0005524 GO:0051082	O
2	Glutamate dehydrogenase	proS	604	66,842	k01881	COG0442	Cytoplasmic	Absence	GO:0006520 GO:0055114 GO:0016491 GO:0016639	J
3	Elongation factor G	fusA	709	78,383	k02355	COG0480	Cytoplasmic	Absence	GO:0006412 GO:0006414 GO:0000166 GO:0003746 GO:0003924 GO:0005525 GO:0005622 GO:0005737	J
4	Transketolase	tkt	702	75,877	k00615	COG0021	Cytoplasmic	Absence	GO:0008152 GO:0003824 GO:0004802 GO:0016740 GO:0046872	G
5	50S ribosomal protein L25	rplY	206	21,815	k02897.	COG1825	Cytoplasmic	Absence	GO:0006412 GO:0003723 GO:0003735 GO:0008097 GO:0019843 GO:0005840 GO:0030529	J
6	Glyceraldehyde 3-phosphate dehydrogenase	BMOU_0196	351	37,881	—	—	Cytoplasmic	Absence	GO:0006006 GO:0055114 GO:0016491 GO:0016620 GO:0050661 GO:0051287	—
7	Glycine-tRNA ligase	glyQS	446	52,295	k01880	COG0423	Cytoplasmic	Absence	GO:0006412 GO:0006418 GO:0006426 GO:0000166 GO:0004812 GO:0004820 GO:0005524 GO:0016874 GO:0046983 GO:0005737	J
8	60 kDa chaperonin	groL	541	56,837	k04077	COG0459	Cytoplasmic	Absence	GO:0006457 GO:0042026 GO:0000166 GO:0005524 GO:0051082 GO:0005737	O
9	Elongation factor Tu	tuf	399	43,936	k02358.	COG0050	Cytoplasmic	Absence	GO:0006412 GO:0006414 GO:0000166 GO:0003746 GO:0003924 GO:0005525 GO:0005622 GO:0005737	J
10	Xylose isomerase	xylA	449	50,765	k01805	COG2115	Cytoplasmic	Absence	GO:0005975 GO:0006098 GO:0042732 GO:0000287 GO:0009045 GO:0016853 GO:0046872 GO:0005737	G
11	ABC transporter substrate-binding protein	BBPC_1795	430	47,488	K10117	COG1653	Cytoplasmic	Absence	GO:0006810 GO:0005215	G
12	Elongation factor Ts	tsf	283	29,835	k02357	COG0264	Cytoplasmic	Absence	GO:0006412 GO:0006414 GO:0003746 GO:0005622 GO:0005737	J
13	Transaldolase	tal	367	39,871	k00616	COG0176	Cytoplasmic	Absence	GO:0005975 GO:0006098 GO:0003824 GO:0004801 GO:0016740 GO:0005737	G
14	ABC transporter substrate-binding protein	BBPC_1231	550	59,251	K15580	COG4166	Cytoplasmic	Absence	GO:0055085 GO:0043190	E
Proteins specific for patients’ intestinal microbiota compared with the normal										
1	Ketol-acid reductoisomerase	ilvC	350	38,768	—	—	Cytoplasmic	Absence	GO:0008652 GO:0009082 GO:0009097 GO:0009099 GO:0055114 GO:0004455 GO:0016491 GO:0016853	—
2	Phosphoglycerate kinase	pgp	401	41,995	k00927	COG0126	Cytoplasmic	Absence	GO:0006096 GO:0016310 GO:0000166 GO:0004618 GO:0005524 GO:0016301 GO:0016740 GO:0005737	G
3	50S ribosomal protein L4	rplD	221	23,761	K02926	COG0088	Unknown	Absence	GO:0006412 GO:0003723 GO:0003735 GO:0019843 GO:0005840 GO:0030529	J
4	Ribose-phosphate pyrophosphokinase	prs	337	36,843	K00948	COG0462	Cytoplasmic	Absence	GO:0006015 GO:0009165 GO:0016310 GO:0000166 GO:0000287 GO:0004749 GO:0005524 GO:0016301 GO:0016740 GO:0046872 GO:0005737	F E
5	Probable thiol peroxidase	tpx	171	18,391	K11065	COG2077	Periplasmic	Absence	GO:0005623 GO:0045454 GO:0055114 GO:0098869 GO:0004601 GO:0008379 GO:0016209 GO:0016491 GO:0016684 GO:0005623	O
6	30S ribosomal protein S4	rpsD	208	23,719	K02986	COG0522	Cytoplasmic	Absence	GO:0006412 GO:0003723 GO:0003735 GO:0019843 GO:0005622 GO:0005840 GO:0015935 GO:0030529	J
7	50S ribosomal protein L3	rplC	213	22,687	K02906	COG0087	Cytoplasmic	Absence	GO:0006412 GO:0003723 GO:0003735 GO:0019843 GO:0005622 GO:0005840 GO:0030529	J

^a^Categories were taken from the TIGR-CMR (www.tigr.org) and the abbreviation was used to mark the categories. *J* Translation, *O* Posttranslational modification, protein turnover, chaperones, *G* Carbohydrate transport and metabolism, *E* Amino acid transport and metabolism, *F* Nucleotide transport and metabolism; —: not in COGs

Compatible with the important sugar degradation potential of the gut microbiota, various proteins involved in carbohydrate transport and metabolism were identified and enhanced in patients, including transketolase, transaldolase, xylose isomerase, and glyceraldehyde 3-phosphate dehydrogenase, which were grouped into the KEGG pathway of map 00030 (Pentose phosphate pathway), map 00040 (Pentose and glucuronate interconversions), map 00051 (Fructose and mannose metabolism), map 00010 (Glycolysis/Gluconeogenesis), as well as map 01110 (Biosynthesis of secondary metabolites). The abundance of bacterial proteins devoted to the utilization of carbohydrates testified for their importance as metabolic substrates in the intestinal tract.

The most significantly differential protein was glyceraldehyde 3-phosphate dehydrogenase (EC1.2.1.12), which catalyzes the oxidative phosphorylation of glyceraldehyde 3-phosphate (G3P) to 1,3-bisphosphoglycerate (BPG) using the cofactor NAD. This protein is involved in step 1 of the subpathway, part of carbohydrate degradation and glycolysis pathway, that synthesizes pyruvate from D-glyceraldehyde 3-phosphate. 3-phosphoglycerate kinase assists in the transformation of 3-phospho-D-glycerate to 3phospho-D-glyceroyl phosphate by consumption of ATP.

Another significantly differential protein was glutamate dehydrogenase (GDH). Moreover, detailed analysis of these peptides revealed GDH to show a high level of redundancy in the intestinal tract since we could identify it as a major protein in a large variety of bacterial families, including *Bacteroidaceae*, *Streptococcaceae*, *Ruminococcaceae* and *Bifidobacteriaceae* (Additional file 1: Table S1). It is known that GDH not only links the nitrogen and the carbon-cycle via the incorporation of ammonia into 2-ketoglutarate, but also have another metabolic role and act as an electron sink [12]. This pathway, which operates in several strict anaerobes to assure a low level of free electrons, resulting in the net conversion of pyruvate and ammonia into alanine while consuming NAD(P)H that can be generated via a ferredoxin NAPDH oxidoreductase [12]. This potential role of intestinal GDH as an electron sink requires the activity of aminotransferases, many of which were identified in the metaproteome, including branched-chain amino acid aminotransferase, aminotransferase class-V family protein, phosphoserine aminotransferase, taurine--pyruvate aminotransferase, aspartate/tyrosine/aromatic aminotransferase, glucosamine--fructose-6-phosphate aminotransferase, acetylornithine aminotransferase, N-succinyldiaminopimelate aminotransferase, histidinol-phosphate aminotransferase, 4-aminobutyrate aminotransferase, which were all specially enhanced in BP and CP patients.

### Unique fecal microbial proteins and metabolism for LC patients

To identify the fecal microbiota proteins specific for patients, we selected proteins that were found only in all the three patients but absent in the normal. A total of seven proteins fulfill the criterion. Charicteristics of unique fecal microbial proteins for LC patients were shown in Table 3.

**Fig. 3:**
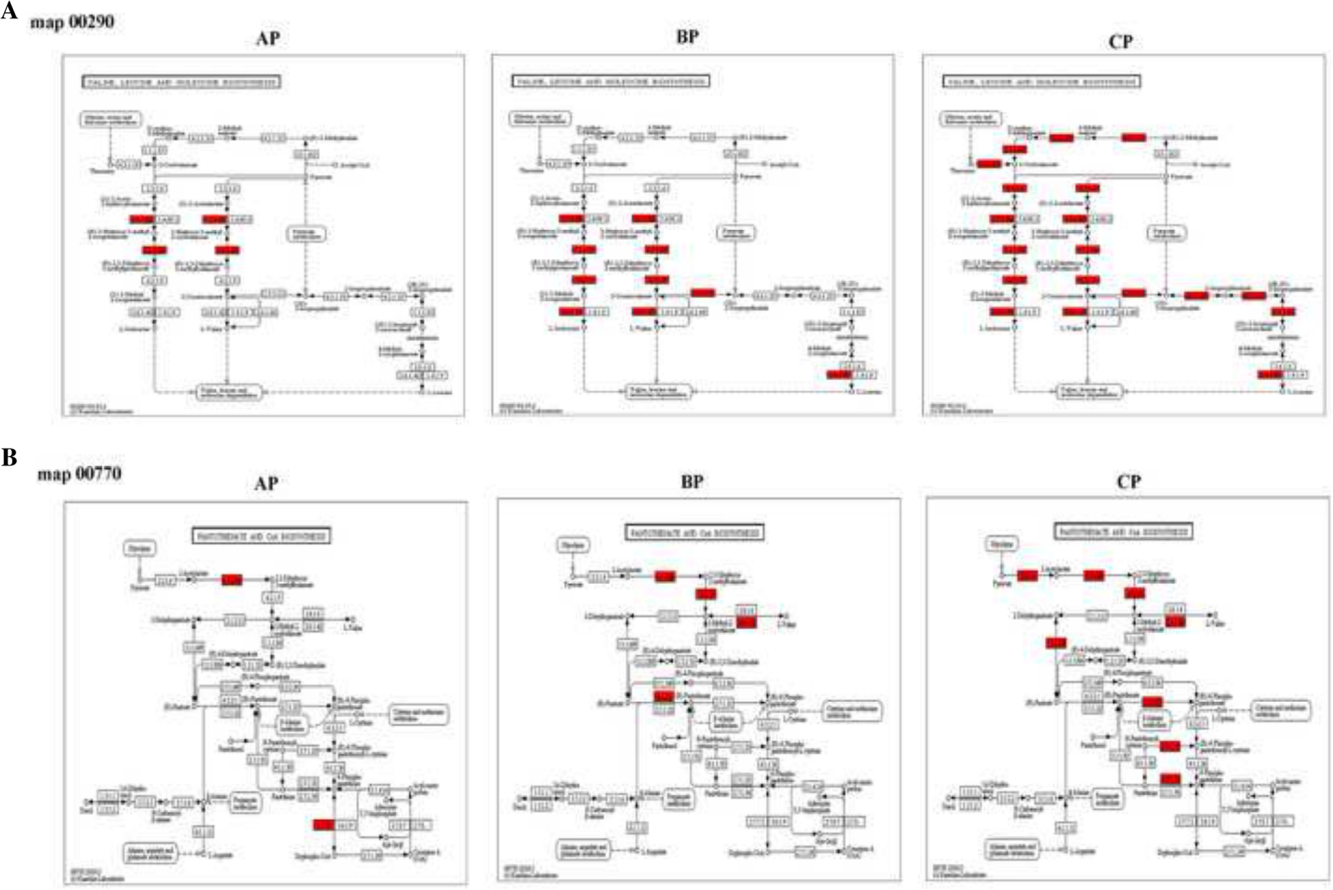
Specific expressed proteins for LC patients allocated to the KEGG pathway of map 00290 (**a**) and map 00770 (**b**). Proteins specific expressed for LC patients were highlighted. As the patients’ condition worse, the number of specific enzymes from the two metabolic pathways were remarkably increased, and the metabolic pathways were enhanced. In patient CP, the specific enzymes from map 00290 and map 00770 were almost covered the whole metabolic pathway

Additionally, Two KEGG pathways, map 00290 (Valine, leucine and isoleucine biosynthesis) and map 00770 (Pantothenate and CoA biosynthesis) were common in the fecal microbiota from the three patients and absent in normal. As the patients’ condition worse, the number of specific enzymes from the two metabolic pathways were remarkably increased, and the metabolic pathways were enhanced. In patient CP, the specific enzymes from map 00290 and map 00770 were almost covered the whole metabolic pathway (Fig. 3). LC patients have an enhanced function of branched-chain amino acid (BCAA) and vitamin metabolism, which were in accordance with the metagenomic results from Wei [21].

The most unique enzymes detected were ketol-acid reductoisomerase (EC 1.1.1.86) and dihydroxy-acid dehydratase (EC 4.2.1.9). Degradation of BCAA involved the branched-chain alpha-keto acid dehydrogenase complex. Those two enzymes were both important in the KEGG pathways of map 00290 and map 00770 and have the protein interactions of neighborhood and coexpression.

Ketol-acid reductoisomerase (EC 1.1.1.86), specific in fecal microbiota from patients, were key enzyme from metabolic pathway of valine, leucine and isoleucine biosynthesis and pantothenate and CoA biosynthesis. This enzyme can catalyse the reduction of 2-ethyl-2-hydroxy-3-oxobutanoate to 2,3-dihydroxy-3-methylpentanoate. This protein is involved in step 2 of the subpathway that synthesizes L-valine from pyruvate and L-isoleucine from 2-oxobutanoate, which are part of the pathway of L-valine biosynthesis and L-isoleucine biosynthesis, respectively. The chemical reactions and pathways resulting in the formation of valine, 2-amino-3-methylbutanoic acid and isoleucine, (2R*, 3R*)-2-amino-3-methylpentanoic acid. In addition, this enzyme is involved in step 2 of the subpathway that synthesizes patothenate from pyruvate, which are part of the pathway of Pantothenate and CoA biosynthesis.

Dihydroxy-acid dehydratase (EC 4.2.1.9), specific in the fecal microbiota from BP and CP, catalyzes third step in the common pathway leading to biosynthesis of branched-chain amino acids and is important in the KEGG pathway of map 00290 and map 00770. This protein is involved in step 3 of the subpathway that synthesizes L-isoleucine from 2-oxobutanoate and L-valine from pyruvate, which are part of the pathway of L-valine biosynthesis and L-isoleucine biosynthesis, respectively.

Branched-chain-amino-acid transaminase (EC 2.6.1.42), specific in the fecal microbiota from BP and CP, belongs to the class-IV pyridoxal-phosphate-dependent aminotransferase family. Branched-chain-amino-acid transaminase can catalyze the transfer of an alpha-amino group from an amino acid to an alpha-keto acid leading to biosynthesis of branched-chain amino acids and is important in the KEGG pathway of map 00290 and map 00770. The amino group is usually covalently bound by the prosthetic group pyridoxal phosphate.

### Non-bacterial proteins

The vast majority of the identified proteins, approximately 87.7 %, were of microbial origin. Moreover, in total, 619 human proteins, excluding possible contaminants [22], were identified as representing host cell activity along the digestive tract. In general, the identified human proteins were mostly involved in carbohydrates and proteins digestion, maintaining mucosal barrier function, and providing energy resources for intestinal microbes.

## Discussion

In any microbiome environment, expression of microbiota proteins were all closely affected by the microenvironment and bacteria-host interactions [23]. However, culture-based research was unable to reappear the real condition of microbiome in the environment and the insufficient microbiome sequence information confounded identification of the proteins [12]. Currently, people were focusing more and more interests on metaproteomics approaches to monitor the disease related functional products of the microbiota [13], and the ongoing protein library analysis enabled meaningful identification in time [2, 24]. For the first time, extraction of metaproteome from LC patients and tentative identification using LC-MS/MS were carried out in this study. To retrieve further functional information, we annotated these proteins based on the GO and KEGG classification and analyzed the characteristic of the intestinal microbiota from LC patients at the expression level.

As revealed by the protein identification searched against the publically available database for Uniprot-Bacteria database, nearly equal proteins were identified from AP and AN, however, almost twice proteins were identified from BP and CP compared with the normal, implying that as the disease processed, the changes in intestinal microenvironment caused by cirrhosis compelled fecal microbiota to enhance their growth activity and protein expression to survival.

A LC-related core metapoteome including 119 proteins was described in this study. Majority of the proteins could be assigned to carbohydrate and amino acid transport and metabolism, reflecting the active metabolic ability of the intestinal microbiota from LC patients. Among those 119 core proteins, 14 proteins, involved in carbohydrate transport and metabolism, enhanced their expression levels in the fecal microbiota from LC patients, which further illustrated that human gut bacteria encountered a broad spectrum of carbohydrate substrates.

Fourteen KEGG pathway maps were detected to have different metabolic capacities in the fecal microbiota between patients and the normal, including eleven pathways enhanced and three pathways weakened in patients. In the same metabolic pathway, different proteins were identified in different samples, suggesting the abundant species diversity and protein complexcity, that is why we got rid of species boundaries and regarded fecal microbiota as a whole in this study.

Seven proteins were identified as the unique fecal microbial proteins for LC patients compared with the normal. Correspondingly, two KEGG pathways, map 00290 (Valine, leucine and isoleucine biosynthesis) and map 00770 (Pantothenate and CoA biosynthesis), were unique in the fecal microbiota from LC patients. More interestingly, as the patients’ condition worse, the patients-specific enzymes were almost covered the whole metabolic pathway. Valine, leucine and isoleucine were all BCAA, which were among the nine essential amino acids for humans. Pantothenate was an essential nutrient for human and used to synthesize CoA, as well as to synthesize and metabolize proteins, carbohydrates, and fats. Pantothenate, in the form of CoA, is also required for acylation and acetylation, which, for example, are involved in signal transduction and enzyme activation and deactivation, respectively [25, 26]. CoA was important in energy metabolism for pyruvate to enter the TCA cycle as acetyl-CoA, and for α-ketoglutarate to be transformed to succinyl-CoA in the cycle, as well as in the biosynthesis of many important compounds such as fatty acids, cholesterol, and acetylcholine [26]. BCAA and pantothenate were both important in a wide array of key biological roles. LC patients were weak fitness and as a result the peripheral tissues need to increase their consumption of a variety of nutrients including carbohydrates, BCAA and vitamins. The specific expression of bacterial proteins devoted to map 00290 and map 00770 testified the compensation for the fragile health and need for nutrition.

## Conclusions

Altogether, we observed a first and highly comparable cirrhosis-specific metaproteome clustering of fecal microbiota. Our findings provided a supportive evidence for the presence of a LC-related substantial metaproteome core which enhanced the expression level involved in sugar transport and degradation, as well as BCAA, pantothenate and CoA biosynthesis. Our results suggested that the fecal microbiota not only had strong adaptability to the intestinal microenvironment, but also could compensate for the fragile and innutritious body of cirrhotic patients.

## Additional files

Additional file 1: Table S1. Protein identification with peptide masses searched against the publically available database for Uniprot-Bacteria database (XLSX 635 kb)
Additional file 2: Table S2. Protein identification with peptide masses searched against the publically available database for Uniprot-Human database (XLSX 50 kb)
Additional file 3: Table S3. Core metaproteome of intestinal microbiota from LC patients (DOCX 27 kb)
Additional file 4: Table S4. Fourteen KEGG pathway maps detected to have different metabolic capacities in the fecal microbiota between patients and the normal (DOC 94 kb)

## Abbreviations

BCAA: Branched-chain amino acid
BMI: Body mass index
CTP: Child-Turcotte-Pugh
DTT: DL-Dithiothreitol
EDTA: Ethylene Diamine Tetraacetie Acid
G3P: Glyceraldehyde 3-phosphate
GDH: Glutamate dehydrogenase
HEPES: 4-(2-Hydroxyethyl)-1-piperazineethanesulfonic acid
IAM: Iodoacetamide
LC: Liver cirrhosis
PMSF: Phenylmethanesulfonyl fluoride
TCA: Trichloroacetic acid
TEAB: Tetraethylammonium bromide
