# Supplementary material for: Cirrhosis related functionality characteristic of the fecal microbiota as revealed by a metaproteomic approach
Source: BMC Gastroenterol. 2016 Oct 4;16:121. doi: 10.1186/s12876-016-0534-0 (PMC5051048; doi:10.1186/s12876-016-0534-0)
Supplement: Additional file 3: Table S3. — Core metaproteome of intestinal microbiota from LC patients (DOCX 27 kb) [file 12876_2016_534_MOESM3_ESM.docx]

**Supplementary table 3. Core metaproteome of intestinal microbiota from LC patients**

| Description | KO | COG | Functional category^a^ |
| --- | --- | --- | --- |
| 10 kDa chaperonin | K04078 | COG0234 | O |
| 2,3-bisphosphoglycerate-dependent phosphoglycerate mutase | K01834 | COG0588 | G |
| 30S ribosomal protein S1 | K02945 | COG0539 | J |
| 30S ribosomal protein S10 | K02946 | COG0051 | J |
| 30S ribosomal protein S13 | K02952 | COG0099 | J |
| 30S ribosomal protein S2 | K02967 | COG0052 | J |
| 30S ribosomal protein S3 | K02982 | COG0092 | J |
| 30S ribosomal protein S4 | K02986 | COG0522 | J |
| 30S ribosomal protein S6 | K02990 | COG0360 | J |
| 50S ribosomal protein L1 | K02863 | COG0081 | J |
| 50S ribosomal protein L10 | K02864 | COG0244 | J |
| 50S ribosomal protein L15 | K02876 | COG0200 | J |
| 50S ribosomal protein L16 | K02878 | COG0197 | J |
| 50S ribosomal protein L18 | K02881 | COG0256 | J |
| 50S ribosomal protein L19 | K02884 | COG0335 | J |
| 50S ribosomal protein L2 | K02886 | COG0090 | J |
| 50S ribosomal protein L20 | K02887 | COG0292 | J |
| 50S ribosomal protein L21 | K02888 | COG0261 | J |
| 50S ribosomal protein L25 | K02897 | COG1825 | J |
| 50S ribosomal protein L3 | K02906 | COG0087 | J |
| 50S ribosomal protein L4 | K02926 | COG0088 | J |
| 50S ribosomal protein L5 | K02931 | COG0094 | J |
| 50S ribosomal protein L6 | K02933 | COG0097 | J |
| 50S ribosomal protein L7/L12 | K02935 | COG0222 | J |
| 60 kDa chaperonin | K04077 | COG0459 | O |
| ABC transporter ATP-binding protein | K02000 K06861 K09972 K09810 | COG4175 COG1137 COG1126COG1136 | E M E M |
| ABC transporter substrate-binding protein | K05772 K02012 K02027 | COG2998  COG1840  COG1653 COG2182 | P P G G |
| Acetate kinase | K00925 | COG0282 | C |
| Acyl carrier protein | K02078 | COG0236 | I Q |
| Adenylosuccinate synthetase | K01939 | COG0104 | F |
| Alanine--tRNA ligase | K01872 | COG0013 | J |
| Alcohol dehydrogenase | K00001 | COG0604 COG1062 COG1064 COG1454 | C R R G C |
| Aldehyde dehydrogenase family protein | K00135 | COG1012 | C |
| Aldehyde-alcohol dehydrogenase | K04072 | COG1012 | C |
| Alkyl hydroperoxide reductase subunit C | K03386 | COG0450 | V |
| Alpha-1,4 glucan phosphorylase | K00688 | COG0058 | G |
| AMP-binding enzyme | K01897 | COG0318 COG1022 | I Q I |
| ATP synthase subunit alpha | K02132 | COG0056 | C |
| ATP synthase subunit beta | K02133 | COG0055 | C |
| ATP-dependent zinc metalloprotease FtsH | K03798 | COG0465 | O |
| Beta-galactosidase | K01190 | COG3250 | G |
| Bifunctional purine biosynthesis protein PurH | K00602 | COG0138 | F |
| Carbamoyl-phosphate synthase large chain | K01955 | COG0458 | E F |
| Cell division protein FtsZ | K03531 | COG0206 | D |
| Chaperone protein DnaK | K04043 | COG0443 | O |
| Chaperone protein HscA | K04044 | COG0443 | O |
| Chaperone protein HtpG | K04079 | COG0326 | O |
| Choloylglycine hydrolase | K01442 | COG3049 | M R |
| DNA gyrase subunit B | K02470 | COG0187 | L |
| DNA-directed RNA polymerase subunit alpha | K03040 | COG0202 | K |
| D-xylulose 5-phosphate/D-fructose 6-phosphate phosphoketolase | K01621 | COG3957 | G |
| Elongation factor G | K02355 | COG0480 | J |
| Elongation factor P | K02356 | COG0231 | J |
| Elongation factor Ts | K02357 | COG0264 | J |
| Elongation factor Tu | K02358 | COG0050 | J |
| Enolase | K01689 | COG0148 | G |
| Fructose-1,6-bisphosphate aldolase, class II | K01624 | COG0191 | G |
| Fumarate reductase, flavoprotein subunit | K00239 | COG1053 | C |
| Fumarate reductase, iron-sulfur subunit | K00240 | COG0479 | C |
| Galactokinase | K00849 | COG0153 | G |
| Gamma-subunit,methylmalonyl-CoA decarboxylase | K18426 | — | — |
| Glucosamine-6-phosphate deaminase | K02564 | COG0363 | G |
| Glucose-6-phosphate isomerase | K01810 | COG0166 | G |
| Glutamate dehydrogenase | K00260 | COG0334 | E |
| Glutamate synthase (NADPH), homotetrameric | K00266 | COG0493 | E R |
| Glutamine synthetase | K01915 | COG0174 | E |
| Glyceraldehyde-3-phosphate dehydrogenase | K00150 K00131 | COG0057  COG1012 | G C |
| Glycine--tRNA ligase | K01880 | COG0423 | J |
| GMP synthase [glutamine-hydrolyzing] | K01951 | COG0518 COG0519 | F F |
| Inorganic pyrophosphatase | K01507 | COG0221 | C P |
| Inosine-5'-monophosphate dehydrogenase | K00088 | COG0516 COG0517 | F I |
| Ketol-acid reductoisomerase | K00053 | COG0059 | E H |
| L-arabinose isomerase | K01804 | COG2160 | G |
| L-lactate dehydrogenase | K00016 | COG0039 | C |
| Malate dehydrogenase | K00024 | COG0039 | C |
| Membrane protein | K01991 | COG1596 | M |
| N utilization substance protein B homolog | K03625 | COG0781 | K |
| NADP-dependent glyceraldehyde-3-phosphate dehydrogenase GapN | K00131 | COG1012 | C |
| Ornithine carbamoyltransferase | K00611 | COG0078 | E |
| Orotate phosphoribosyltransferase | K00762 | COG0461 | F |
| Oxidoreductase | K00118 | COG0673 | R |
| Phosphoenolpyruvate carboxykinase [ATP] | K01610 | COG1866 | C |
| Phosphoenol pyruvate-protein phosphotransferase | K08483 | COG1080 | G |
| Phosphoglucomutase | K01835 | COG0033 | G |
| Phosphoglycerate kinase | K00927 | COG0126 | G |
| Phosphoketolase | K01621 | — | — |
| Phosphoribosylaminoimidazole-succinocarboxamide synthase | K01923 | COG0152 | F |
| Phosphoribosylformylglycinamidine cyclo-ligase | K01933 | COG0150 | F |
| Phosphoribosylformylglycinamidine synthase | K01952 | COG0046 COG0047 | F F |
| Polyribonucleotide nucleotidyltransferase | K00962 | COG1185 | J |
| Probable thiol peroxidase | K11065 | COG2077 | O |
| Protein RecA | K03553 | COG0468 | L |
| Protein translocase subunit SecA | K03070 | COG0653 | U |
| Putative elongation factor Tu-like protein |  |  |  |
| Putative lipoprotein | K05811 | COG5544 | S |
| Putative methylmalonyl-CoA mutase | K01847 | — | — |
| Putative S-layer-like domain protein | — | — | — |
| Pyruvate kinase | K00873 | COG0469 | G |
| Pyruvate-flavodoxin oxidoreductase | K03737 | COG0674 COG1013 COG1014 | C C C |
| Ribose-phosphate pyrophosphokinase | K00948 | COG0462 | F E |
| Ribosomal protein S1 | K02945 | COG0539 | J |
| Ribosomal protein S3 | K02982 | COG0092 | J |
| Ribosome-binding ATPase YchF | K06942 | COG0012 | J |
| Ribosome-recycling factor | K02838 | COG0233 | J |
| S-adenosylmethionine synthase | K00789 | COG0192 COG1812 | H H |
| Aspartate-semialdehyde dehydrogenase | K00133 K15786 | COG0136 COG1012 | E C |
| Sugar ABC transporter substrate-binding protein | K02027 | COG1653 COG2182 | G G |
| Threonine--tRNA ligase | K01868 | COG0441 | J |
| Transaldolase | K00616 | COG0176 | G |
| Transcription elongation factor GreA | K03624 | COG0782 | K |
| Transcription termination/antitermination protein NusG | K02601 | COG0250 | K |
| Transketolase | K00615 | COG0021 | G |
| Trigger factor | K03545 | COG0544 | O |
| Triosephosphate isomerase | K01803 | COG0149 | G |
| Tryptophanase | K01667 | COG3033 | E |
| UDP-glucose 4-epimerase | K01784 | COG1087 | M |
| Uronate isomerase | K01812 | COG1904 | G |
| Xylose isomerase | K01805 | COG2115 | G |
| Xylulose-5-phosphate/fructose-6-phosphate phosphoketolase | K01621 | — | — |

^a^ Abbreviation of cellular role categories. Categories were taken from the TIGR-CMR (www.tigr.org) and the abbreviation was used to mark the categories. J, translation; K, transcription; L, replication, recombination, and repair; D, cell cycle control, mitosis, and meiosis; V, defense mechanisms; M, cell wall/membrane biogenesis; U, intracellular trafficking and secretion; O, post-translational modification, protein turnover, chaperones; C, energy production and conversion; G, carbohydrate transport and metabolism; E, amino acid transport and metabolism; F, nucleotide transport and metabolism; H, coenzyme transport and metabolism; I, lipid transport and metabolism; P, inorganicion transport and metabolism; Q, secondary metabolite biosynthesis, transport, and catabolism; R, general function prediction only; S, function unknown; —, not in Clusters of Orthologous Groups (COG).
