# Supplementary material for: Cirrhosis related functionality characteristic of the fecal microbiota as revealed by a metaproteomic approach
Source: BMC Gastroenterol. 2016 Oct 4;16:121. doi: 10.1186/s12876-016-0534-0 (PMC5051048; doi:10.1186/s12876-016-0534-0)
Supplement: Additional file 4: Table S4. — Fourteen KEGG pathway maps detected to have different metabolic capacities in the fecal microbiota between patients and the normal (DOC 94 kb) [file 12876_2016_534_MOESM4_ESM.doc]

**Supplementary table 4. Fourteen KEGG pathway maps detected to have different metabolic capacities in the fecal microbiota between patients and the normal.**

| **Pathway**  **Maps** | **Pathway Name** | **Group** | **Protein in Background^a^** | **Protein in**  **Diff Exp^b^** | **Protein List^c^** |
| --- | --- | --- | --- | --- | --- |
| **Enhanced KEGG pathway maps in fecal microbiota from patients** | | | | | |
| map03018 | RNA degradation | A | 68 | 23 | L7VN33, R6G3P0, Q93TK9, V5QQV9, A9KKU0, Q1L3Y6, E5VVZ5, W4N8D8, D4M4G2, Q1L3T7, Q8G879, Q8KHD0, S2ZIX3, Q9L6E9, C0BVC8, A1BET8, B2KAX0, C4ZD46, C4Z3R4, C4Z1J4, N2B4E7, E4SS36, E4SPQ6 |
|  |  | B | 134 | 9 | R6J7H5, L7EKC7, R6WZJ8, D4VB39, D4M4G2, B2KAX0, C4ZD46, D4V7T4, C0BV87 |
|  |  | C | 87 | 10 | D1NU71, A0A083X4Y5, V5QQV9, A0A083X6R1, A0A083WXV5, A1A008, Q8G879, A0A083X0Y6, C3K1A0, C0BV87 |
| map00910 | Nitrogen metabolism | A | 52 | 20 | N2A5B5, E5VZ78, A0A083WXC9, A0A083X1F7, A0A083WXG1, D4M5L5, F1TGI8, D4M625, A0A083X144, A0A083X6R1, A0A083X732, A0A083X541, A0A083WW18, N2AGL8, A0A083WWX5, R5EQH1, Q9K580, W1UJZ3, Q4ZH57, A0A083X2E0 |
|  |  | B | 31 | 2 | B6XSI8, A0A087BKN1 |
|  |  | C | 31 | 2 | B6XSI8, A0A087BKN1 |
| map00051 | Fructose and mannose metabolism | A | 33 | 10 | C0BQ37, D4M495, Q39U97, C5EP52, E5VYZ0, D4M500, B1QYZ6, D4M3C9, D4IZD6, C5EJ71 |
|  |  | B | 71 | 14 | U5C951, R6JN11, C5EP52, E5VYZ0, A0A078S0A4, A6LMG2, Q8A9M2, D4VCT5, R6R773, Q8G3Q1, A0A076IW67, Q1A689, D4V5Y1, A6L792 |
|  |  | C | 39 | 2 | A1A0H0, W4N7E9 |
| map02020 | Two-component system | A | 52 | 16 | N2A5B5, D4J1B0, A0A083WXC9, A0A083X1F7, A0A083WXG1, A0A083X144, A0A083X6R1, A0A083X732, A0A083X541, N2AD89, A0A083WW18, A0A083WWX5, Q216X5, V2Z3W3, Q4ZH57, A0A083X2E0 |
|  |  | B | 17 | 2 | R6Q1B5, Q216X5 |
|  |  | C | 41 | 2 | F2KB61, A0A087BKN1 |
| map00250 | Alanine, aspartate and glutamate metabolism | A | 56 | 22 | N2A5B5, E5VZ78, A0A083WXC9, A0A083X1F7, A0A083WXG1, C4ZEK2, D4M5L5, F1TGI8, D4M625, A0A083X144, A0A083X6R1, A0A083X732, A0A083X541, A0A083WW18, N2AGL8, A0A083WWX5, R5EQH1, Q9K580, W1UJZ3, C4Z9C9, Q4ZH57, A0A083X2E0 |
|  |  | B | 42 | 4 | B6BKM6, D4V559, D4M625, D4VC17 |
|  |  | C | 47 | 2 | B6XSI8, A0A087BKN1 |
| map00330 | Arginine and proline metabolism | A | 61 | 24 | N2A5B5, N2BC74, B9W5Q0, A0A083WXC9, A0A083X1F7, A0A083WXG1, A4E7H3, D4M466, D4M5L5, F1TGI8, K5IM29, A0A083X144, A0A083X6R1, A0A083X732, A0A083X541, F4LUC8, A0A083WW18, N2AGL8, A0A083WWX5, R5EQH1, Q9K580, C4Z9C9, Q4ZH57, A0A083X2E0 |
|  |  | B | 41 | 7 | D4VCC6, B6BKM6, R6PYU5, C8WG92, D4M5P0, D4VC17, A0A076IQB5 |
|  |  | C | 51 | 2 | B6XSI8, A0A087BKN1 |
| map00230 | Purine metabolism | A | 52 | 29 | Q8G5A9, W4NB83, D4IYK5, B9W5Q0, B7GN89, N2BAU9, W4N8R7, C5EVQ4, B9MQG5, C0BSW4, D4M043, N2B5S4, R6G3P0, W4N8J1, W4N7J9, I1ZLR8, W8TCF3, W4N9D9, R6G7H2, R6G8D8, S2ZIX3, A0A074J1U0, R6PTB0, W4N8Z3, S3A0W1, S2ZD40, D4M4B3, A0A095ZTL0, E5VQD8 |
|  |  | B | 102 | 3 | A0A076J380, C0BSW4, A0A076ILF6 |
|  |  | C | 78 | 2 | D1NUW0, A1A008 |
| map00030 | Pentose phosphate pathway | A | 31 | 15 | C0BQT7, B7GN89, D4M495, J5HYZ0, D4M189, D4M043, G0G082, I1ZLR8, B7GQV6, A0A087BIL4, C0BTI1, C0BTI0, C4ZF71, D4IZD6, C5EJ71 |
|  |  | B | 47 | 5 | R6PYU5, A0A076IW67, C0BTI1, C0BTI0, A0A076IQB5 |
|  |  | C | 47 | 5 | A1A0A9, W4NAP9, W4N6V8, B7GQV5, B7GQV6 |
| map00520 | Amino sugar and nucleotide sugar metabolism | A | 21 | 7 | C0BQT7, B7GN89, F7LNR0, B6XVP7, D4M043, I1ZLR8, B7GUI0 |
|  |  | B | 46 | 2 | D4VCW3, D4V5F5 |
|  |  | C | 36 | 3 | W4N6V8, C0BQK5, C0BTX3 |
| map00040 | Pentose and glucuronate interconversions | A | 17 | 5 | C0BQ37, C0BV07, R6G4Q0, N2A8J2, C4Z5P8 |
|  |  | B | 27 | 8 | U5C951, R6JN11, R6PYU5, Q8A9M2, A0A076J058, Q8G3Q1, A0A076IQB5, A6L792 |
|  |  | C | 24 | 2 | A1A0H0, W4N7E9 |
| map00010 | Glycolysis / Gluconeogenesis | A | 115 | 47 | G2IFW5, C0BQT7, N2ACM8, U5L3V6, C5EUN1, B7GN89, O83023, I1ZJH3, D4M495, O09460, W4NAP8, E5VYY8, Q59309, D4M4Z9, Q39U97, A0A099IC82, R7GE60, E5VYZ0, C0BSW4, J6GY59, D4M043, T4H7R5, A0A087BI29, C4Z0Q6, N2AHQ0, R6G904, V5QQV9, D4M342, I1ZLR8, T8T8X0, R2RV46, D4M500, W4N8D8, D5EVN2, I1ZP68, A0A074J1U0, F5VWF7, Q88YH6, B1QYZ6, D4V441, R6Q0N3, D4IZD6, F9D663, D4J2A7, C4ZBL1, C0BQI3, C5EJ71 |
|  |  | B | 195 | 27 | U5L3V6, R6J7H5, W2C8V9, L7EKC7, W4NAP8, Q59309, D4M4Z9, R7GE60, E5VYZ0, A0A078S0A4, C0BSW4, J6GY59, A6LMG2, R5IMN3, R5IQ16, D4VB39, A6L050, R6R773, A0A076IW67, D4VAQ0, Q88YH6, D4V441, D5ETL5, D4V5Y1, D4V3W4, C4ZBL1, A6LFQ4 |
|  |  | C | 108 | 6 | U5L3V6, B7GQC0, W4NAP8, V5QQV9, W4N6V8, C0BTX3 |
| **Weakened KEGG pathway maps in fecal microbiota from patients** | | | | | |
| map04626 | Plant-pathogen interaction | A | 87 | 38 | A7I3U7, C0BPQ2, D4J1B0, Q5FCW3, K9CI62, N2BAU2, T2W7Q1, N2A8G2, Q47LJ1, C5ELF2, W7CSY8, C4ZB99, B6D3H6, P42480, U5A8P4, W0FL29, Q5HBB0, C4Z2R9, D4VB66, Q6ACZ0, I1ZMY8, Q88VE0, Q8XFP8, Q3A9R3, Q6CZW6, K0AZP3, V8IB70, S6G4J6, D4M4C8, G2MWZ3, V2Z3W3, U7V0J1, C7GZQ3, Q2LQA3, A0A074J0H1, A3UDB2, X8K161, D4J017 |
|  |  | B | 110 | 12 | R5CN47, A0A076J1Z5, E1KT74, D5EUX5, F9D151, D4VB66, Q8A463, K0E7M8, R6W0N2, Q8G5B7, E5VVF3, X8K161 |
|  |  | C | 71 | 4 | C3K2X8, Q6ACZ0, D5MK17, Q8G5B7 |
| map00710 | Carbon fixation in photosynthetic organisms | A | 70 | 28 | G2IFW5, R7EPD3, N2ACM8, C5EUN1, O83023, D4M495, O09460, C0BSZ4, D4M4Z9, Q39U97, E5VYZ0, C0BSW4, D4M189, G0G082, D4M2Q4, C4Z0Q6, P22983, N2A828, D4M500, B7GQV6, D5EVN2, D4J1X9, C0BTI1, A0A074J1U0, B1QYZ6, R6Q0N3, D4IZD6, C4ZBL1 |
|  |  | B | 102 | 19 | D4M4Z9, E5VYZ0, A0A078S0A4, C0BSW4, A6LMG2, W2C1U3, D4M2Q4, R5IMN3, R6R773, A0A076IW67, C0BTI1, D4VAQ0, Q11QQ3, D4V4Y9, D4V5Y1, D4V5Y5, D4V3W4, C4ZBL1, A6LFQ4 |
|  |  | C | 75 | 4 | A1A0A9, W4NAP9, B7GSW8, B7GQV6 |
| map03010 | Ribosome | A | 123 | 49 | P59179, G2KV20, R6G6P9, E5VVG0, E5VVJ2, C0BQS5, C5EVN8, W4N8P5, C0BQR7, A0A074IVY7, C5EW17, C5EW16, E5VUK6, C4ZBD5, C0BSW0, A0A074IW53, D4M0T9, E5VUN8, W4N6P6, Q8G5Z8, A0A087BF58, C4Z9Z6, I1ZLP6, D4M5P6, W4N6L7, C4Z2T0, C4Z2T8, C4Z2R3, Q8G444, Q8G443, F3P8D9, W4N742, W4N741, C4ZBS6, C4ZBS5, Q8G414, C4ZBU5, C4ZBU3, K7A5W7, W4N883, N2BKP4, R6GF89, A0A074IVE3, N2FKN8, C0BQM8, R6C7J8, D4J021, W4N898, C0BQH9 |
|  |  | B | 84 | 7 | C0BT56, W4N8P5, R6JP97, D4M5P6, D4VEA6, D4J021, W2C5V4 |
|  |  | C | 85 | 5 | C0BT56, C0BUS5, W4N6W8, F3P8D9, C0BQH9 |

^a^: Protein in Background: Number of proteins identified and belonged to the metabolic pathway

^b^: Protein in Diff Exp: In the metabolic pathway, number of differential proteins identified between patients and the normal

^c^: Protein List: Differential proteins in the metabolic pathway.

In the same metabolic pathway, differential proteins identified in more than one samples were highlighted.
